# Perceptions of research participation among underrepresented groups: Insights using freelisting methodology

**DOI:** 10.1371/journal.pone.0351215

**Published:** 2026-07-01

**Authors:** Tamar Klaiman, Jasmine A. Silvestri, Emma Britez Ferrante, Dorothy Sheu, Adina Lieberman, Erich Dress, Modele O. Ogunniyi, Neal W. Dickert, Meghan B. Lane-Fall, Rosemary Frasso, Rachel Kohn

**Affiliations:** 1 Behavioral Economics to Transform Trial Enrollment Representativeness (BETTER) Center, American Heart Association Strategically Focused Research Network on the Science of Diversity in Clinical Trials, Philadelphia, Pennsylvania, United States of America; 2 Palliative and Advanced Illness Research (PAIR) Center, Perelman School of Medicine at the University of Pennsylvania, Philadelphia, Pennsylvania, United States of America; 3 Department of Medical Ethics and Health Policy, Perelman School of Medicine, University of Pennsylvania, Philadelphia, Pennsylvania, United States of America; 4 Center for Health Incentives and Behavioral Economics (CHIBE), Perelman School of Medicine at the University of Pennsylvania, Philadelphia, Pennsylvania, United States of America; 5 Department of Medicine, Division of Cardiology, Emory University School of Medicine, Atlanta, Georgia, United States of America; 6 Grady Health System, Atlanta, Georgia, United States of America; 7 Department of Epidemiology, Emory University Rollins School of Public Health, Atlanta, Georgia, United States of America; 8 Department of Anesthesiology, Columbia University Vagelos College of Physicians and Surgeons, New York, New York, United States of America; 9 Jefferson College of Population Health, Thomas Jefferson University, Philadelphia, Pennsylvania, United States of America; 10 Asano-Gonnella Center for Research in Medical Education and Health Care, Sidney Kimmel Medical College, Thomas Jefferson University, Philadelphia, Pennsylvania, United States of America; 11 Department of Medicine, Perelman School of Medicine at the University of Pennsylvania, Philadelphia, Pennsylvania, United States of America; 12 Leonard Davis Institute of Health Economics, Perelman School of Medicine at the University of Pennsylvania, Philadelphia, Pennsylvania, United States of America; Sreenidhi Institute of Science and Technology, INDIA

## Abstract

**Background:**

Low enrollment and retention in clinical research disproportionately impact Black, Hispanic or Latinx, women, and rural populations, undermining generalizability and perpetuating health disparities. However, few studies have compared mechanisms driving underrepresentation across populations. Freelisting is a qualitative methodology that elicits lists of terms, explores perspectives about domains, and identifies common themes within groups with shared characteristics; however, it has not been systematically applied to understand research participation across underrepresented populations.

**Objective:**

To explore perspectives on clinical research participation across underrepresented populations using freelisting methodology to ultimately inform culturally-responsive recruitment strategies.

**Methods:**

We conducted a web-based freelisting survey among adults who identified as Black, Hispanic or Latinx, women, and/or resided in rural communities between May and September 2023 across the Philadelphia, Atlanta, and Washington, DC metro areas. Participants listed words or phrases that came to mind in response to three prompts about research and participation. Using Anthropac software, we calculated salience indices to assess the relative importance of terms within and across the underrepresented groups. Terms were categorized by sentiment (positive, neutral, negative) and examined by demographic group and prior research experience.

**Results:**

Of 101 participants (56% Black, 23% Hispanic or Latinx, 80% women, 46% rural), several salient terms were shared, including ‘study,’ ‘knowledge,’ ‘search,’ and ‘scary.’ Sentiment regarding being approached for research was generally positive. In contrast, sentiment about becoming a participant varied, with more negative terms among those never previously invited to join research. ‘Research misconduct’ emerged as uniquely salient among Black participants. Individuals with prior research experience conveyed more positive sentiments overall.

**Conclusions:**

Underrepresented populations hold positive and negative views about clinical research, with more negative perceptions among those never previously approached. These findings suggest that proactive outreach to individuals who have never previously been approached, combined with efforts to address persistent negative perceptions such as fear, may be among the most impactful strategies for improving research representativeness. Future work is needed to understand the contextual information surrounding the sentiments we found, and to elucidate the mechanisms underlying these sentiments, ultimately enabling the development of more effective, culturally-responsive recruitment and retention strategies across diverse groups.

## Background

Advances in clinical medicine are hindered by low enrollment and retention in prospective clinical research, with persistently modest engagement rates across diverse populations [1–8]. These challenges disproportionately impact individuals who identify as Black, Hispanic or Latinx, women, and those residing in rural communities, populations who also have disproportionately higher rates of chronic disease incidence, decreased use of evidence-based treatments, and worse outcomes across disease processes [9–19]. The resulting lack of diversity in study populations undermines the generalizability and public health impact of scientific discoveries, perpetuates health disparities across conditions, and may contribute to mistrust of biomedical research and healthcare systems [1]. Despite the persistence of this deficit [2–5,20], and prior literature exploring barriers and facilitators surrounding prospective research participation among specific underrepresented populations [7,12,13], few studies have explored mechanisms driving underrepresentation across multiple populations in greater depth.

Freelisting is a methodology that offers a structured approach to rapidly explore emotions and perspectives about complex domains and identify common concerns or priorities within a group with shared characteristics [21–24]. This qualitative technique extracts responses in participants’ own words by eliciting lists of elements of a particular domain. The method assumes shared cultural beliefs will produce shared concepts among members of each group, thus allowing researchers to draw comparisons between groups. Freelisting data are not influenced by interview technique and allow insight into respondent priorities without explicitly asking. However, this methodology has not been systematically applied to understand barriers and facilitators to prospective clinical research participation across underrepresented populations.

Therefore, we conducted a web-platform-based freelisting survey among adults who identified as Black, Hispanic or Latinx, women, and/or resided in rural communities. Our objective was to explore emotions and perspectives on prospective clinical research participation across and between underrepresented populations, to ultimately inform the development of more effective, culturally-responsive recruitment and retention strategies across groups.

## Methods

### Design, participants, and setting

We recruited participants ≥18 years who identified as Black, Hispanic or Latinx, women, and/or resided in rural communities between May and September 2023, across five health systems in the Philadelphia, Atlanta, and Washington, DC metro areas, and one community-based organization serving Hispanic or Latinx patients in Philadelphia. We distributed flyers with QR codes that linked to our online Qualtrics [25] survey in primary care, cardiovascular, pulmonary, and radiology clinics, and emergency department waiting rooms. All study materials were available in English and Spanish; Spanish versions were professionally translated.

Participants first completed an 8-question demographic eligibility questionnaire; eligible participants were then directed to an informed consent form. Participants had the option to download a copy of the consent form, and they were required to check a box acknowledging their consent before proceeding to the survey. We continued recruitment until there were ≥20 complete responses for each group of interest, a recommended sample size to calculate salience, i.e., the importance of each term or phrase within and among each group of interest [21–24]. Participants were offered a $25 incentive for completing the survey. The study was approved by the University of Pennsylvania Institutional Review Board (protocol #851603) in accordance with the Declaration of Helsinki.

The freelisting survey was part of a larger survey exploring barriers and facilitators to research participation and included multiple-choice and open-ended questions. It was developed by an interdisciplinary team comprised of clinician-researchers (MLF, RK) and research staff with expertise in qualitative and mixed methods, public health, health equity, community engagement, and prospective research (TK, JAS, EBF, DS, AL). For the freelisting portion of the survey, we first explained the concept of freelisting. We subsequently provided an example question and potential responses unrelated to the survey topics, and we provided the following instructions and prompts (S1 Appendix):

**Instructions:**

1. For each topic, type words and phrases as soon as you think of them.
2. Type ALL the words and phrases you can think of.
3. Type all the words or phrases in the order that you think of them.

Do not edit the order.

1. Type as many words and phrases as you want.
There are no right or wrong answers.

**List the terms or phrases that came to mind in response to each of 3 phrases:**

- Think about the term ‘research.’ What are all the words and phrases that you think of?
- Think about times you were asked to participate in research. Type all the words and phrases to describe how it made you feel.
- Think about being a participant in a research study. What are all the words or phrases that come to mind?

In a traditional freelisting interview, the researcher would actively probe for additional responses to ensure a complete list was obtained for each participant. To mimic this in an online survey, as participants proceeded through the survey, a follow-up question solicited additional terms after the initial responses were submitted. First developed by anthropologists, and employed during short interviews, research has shown that web-based administration can produce comparable results to interviews [24].

### Data cleaning and analysis

Following guidelines described by Keddem et al., three investigators (TK, JAS, EBF) collaboratively cleaned the raw dataset by grouping similar terms and concepts under ‘parent’ terms. For example, the parent term ‘study’ included nested words and phrases such as ‘studying,’ ‘some sort of study,’ ‘project,’ and ‘study of [something specific].’ For each question, words with the same root, synonyms, and similar concepts were combined. Parent terms thus replaced nested terms throughout the dataset. During this process, we created a dictionary of terms from the raw data that were nested under each parent term. A fourth investigator (RF) with extensive expertise in freelisting, provided guidance throughout this process and reviewed our final data dictionary and cleaned dataset (S2 Appendix).

**Fig 1:**
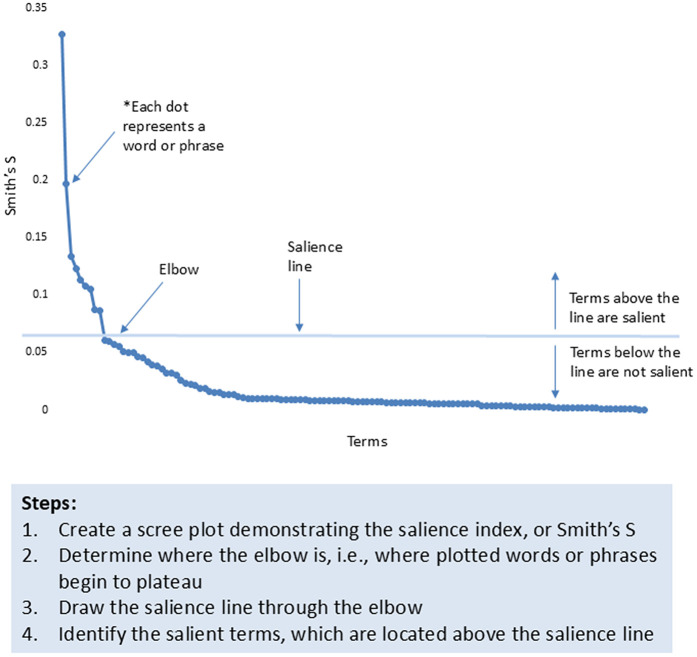
Example scree plot of the salience index (Smith’s S) to determine the salience of freelisting words and phrases in response to a prompt such as ‘Think about the term ‘research’. What are all the words and phrases that you think of?’‌‌.

Cleaned datasets were exported into Visual Anthropac software 1.0, Version 4.98 [26] to determine the salience index scores (Smith’s S) for each term, i.e., the statistic utilized for freelisting analysis. The salience index incorporates the frequency and order of words and phrases across participants as well as the length of the lists:


$$\begin{array}{c} Smith^{\prime }s\;S=\frac{Sum\;of\;the\;word^{\prime }s\;or\;phrase^{\prime }s\;\%\;ranks}{Total\;number\;of\;lists}=\;\frac{\sum \left(\frac{L-R_{j}}{L}\right)}{N} \end{array}$$

where, L is the length of each list, R_j_ is the rank of word or phrase j in the list, and N is the number of lists in the sample. We then examined the salience indices across words and phrases using scree plots (e.g., Fig 1). Four team members (TK, JS, EBF, ED) reviewed each plot and identified the ‘elbow,’ above which terms were considered salient, while those below the elbow were considered not salient by convention [21,22].

We compared salient terms in each group of interest and identified commonalities and unique terms across groups. We also compared those who reported having previously participated in research to those who had never been asked to participate. Additionally, we used a consensus approach (TK, JAS, EBF) to categorize salient terms by sentiment (i.e., positive, neutral, negative). Participants who identified with multiple populations of interest were included in each group’s analysis.

## Results

**Fig 2:**
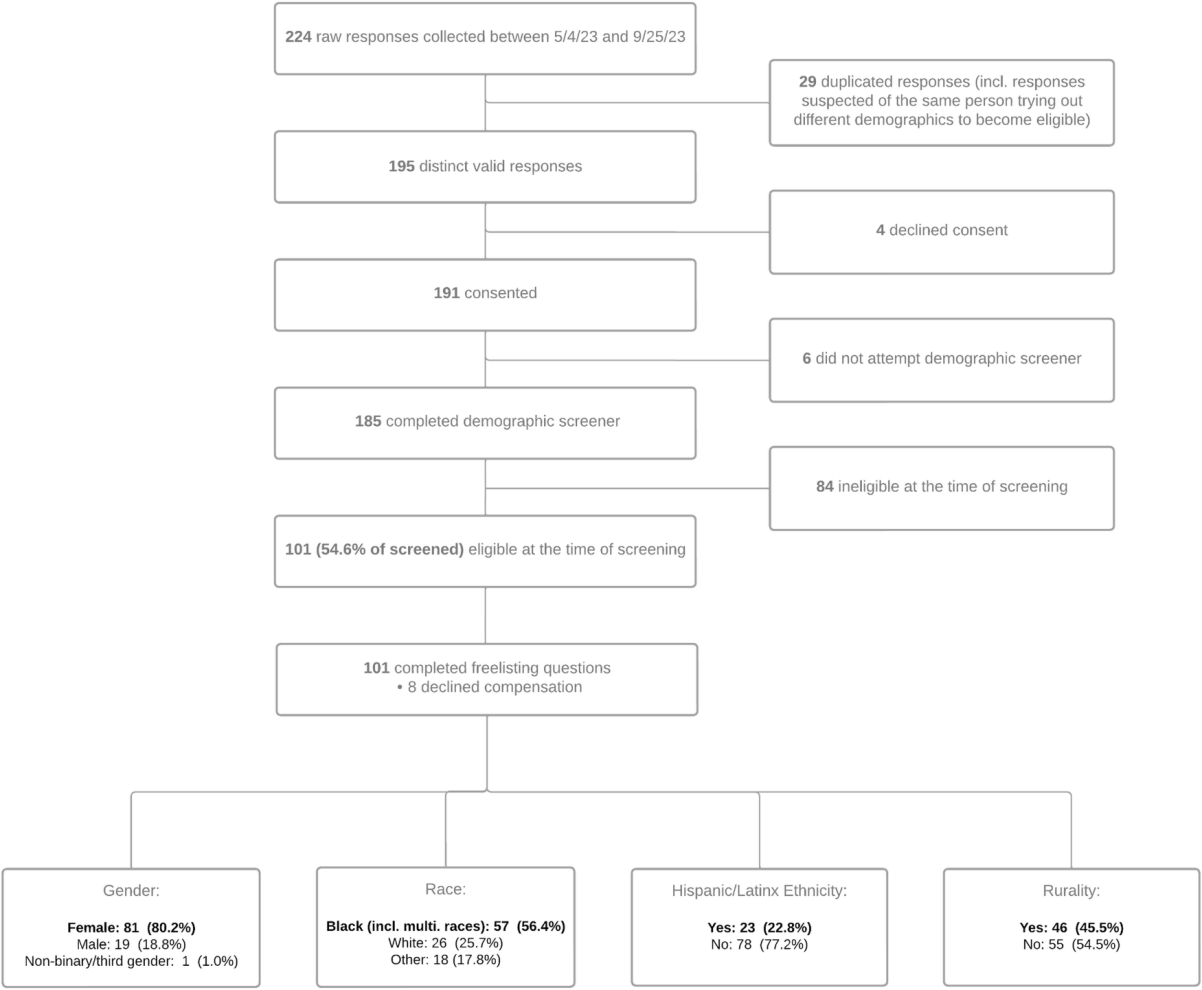
Freelisting survey CONSORT diagram.

Among 195 people who completed the screening step, 4 people declined to consent, 6 did not complete the demographic screen, 84 were ineligible, and 3 did not complete the survey. In total, 101 (55% of those screened) participants completed the freelisting questions. Of these, 57 (56%) identified as Black, 23 (23%) as Hispanic or Latinx, 81 (80%) as women, and 46 (46%) as residing in rural communities. The median age of all participants was 38 years (interquartile range 31–52); 79 (78%) participants identified as belonging to multiple groups; 32 (32%) participants previously participated in research; and 59 (58%) had not previously been asked to participate (Fig 2). Results are presented by survey question.

### Think about the term ‘research.’ What are all the words and phrases that you think of? (S3 Appendix)

**Fig 3:**
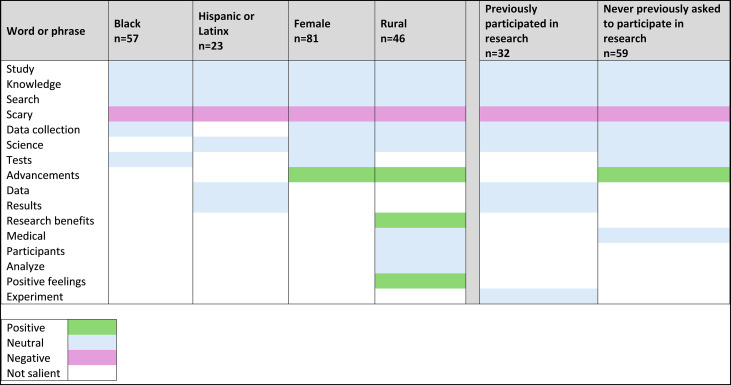
Sentiment comparison for ‘Think about the term ‘research’. What are all the words and phrases that you think of?’.

All populations shared the following salient terms: ‘study,’ ‘knowledge,’ ‘search,’ and ‘scary,’ indicating variability in research perceptions across all groups. The words ‘data’ and ‘results’ were salient exclusively for Hispanic or Latinx participants. ‘Research benefits,’ ‘medical,’ ‘participants,’ ‘analyze,’ and ‘positive feelings’ were salient for rural participants. Black and women participants did not express unique salient terms compared to the other populations. ‘Data,’ ‘results,’ and ‘experiment’ were uniquely salient to those who had previously participated in research, compared to those who had never previously been asked to participate in research, for whom ‘tests,’ ‘advancements,’ and ‘medical’ were salient. Most salient words or phrases had neutral sentiment across groups. The term ‘scary’ was the only salient term with a negative sentiment, but it was salient for all response groups (Fig 3).

### Think about times you were asked to participate in research. Type all the words and phrases to describe how it made you feel. (S4 Appendix)

All populations shared the following salient words and phrases: ‘scary,’ ‘effort,’ ‘never participated,’ ‘curiosity,’ ‘positive feelings,’ ‘being included,’ ‘feeling valued,’ ‘willing to participate,’ and ‘research benefits.’ Black participants had the highest number of unique salient words and phrases. Hispanic or Latinx respondents identified ‘experiment’ and ‘knowledge’ as salient; rural participants ‘important’ and ‘search;’ and women participants, ‘lab rats’ and ‘questions about the project.’

‘Effort,’ ‘important,’ ‘knowledge,’ ‘experiment,’ and ‘search’ were salient among those who had previously been asked to participate in research, of which only ‘effort’ had negative sentiment. ‘Never participated,’ ‘incentives,’ and ‘questions about the project’ were salient terms among those who had never been asked to participate.

**Fig 4:**
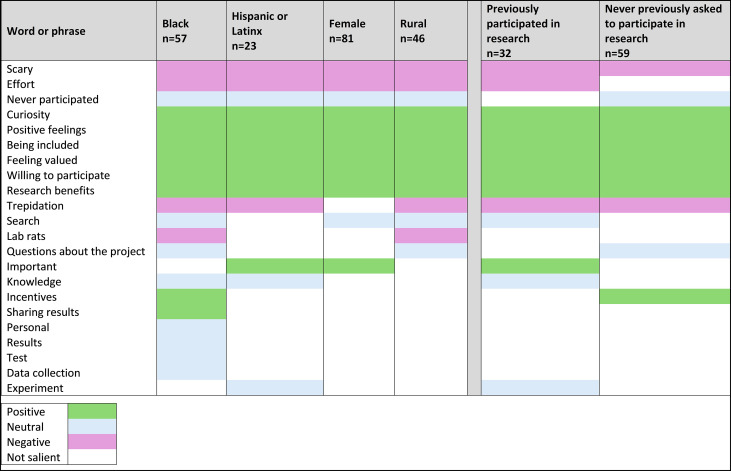
Sentiment comparison for ‘Think about times you were asked to participate in research. Type all the words and phrases to describe how it made you feel’.

Most salient words and phrases exhibited positive sentiment across groups (Fig 4).

### Think about being a participant in a research study. What are all the words or phrases that come to mind? (S5 Appendix)

**Fig 5:**
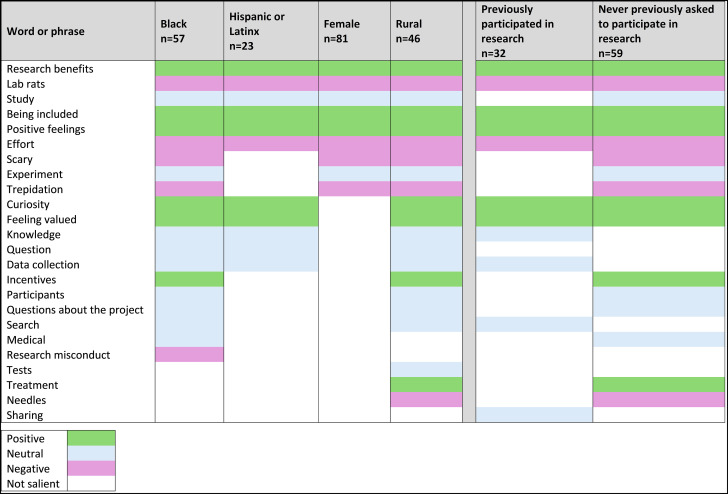
Sentiment comparison for ‘Think about being a participant in a research study. What are all the words or phrases that come to mind?’.

This question yielded the greatest number of salient words and phrases across the questions. ‘Research misconduct’ was uniquely salient for Black respondents. women respondents had numerous salient terms, including ‘tests,’ ‘treatment,’ and ‘needles.’ Hispanic or Latinx and rural participants did not have any unique salient terms. ‘Knowledge,’ ‘data collection,’ ‘search,’ and ‘sharing’ were salient for those who had previously participated in research, while ‘incentives,’ ‘participants,’ ‘questions about the project,’ ‘medical,’ ‘treatment,’ and ‘needles’ were salient for those who had never previously been asked to participate in research. Sentiment varied across the salient words and phrases from positive to neutral to negative; sentiments were similar across groups, with more negative salient terms among those who had never previously been asked to participate in research compared to those who had previously participated (Fig 5).

## Discussion

In this study, we leveraged freelisting to explore emotions and perspectives on research participation among populations underrepresented in prospective research to understand how we can develop more effective, culturally-responsive recruitment and retention strategies across groups. Overall, participants had neutral feelings about the term ‘research’ itself. However, when asked about times they had been asked to participate in research, the sentiment was overwhelmingly positive across groups. When asked about being a research study participant, salient terms spanned the spectrum of sentiments from positive to neutral to negative, similarly across groups, with more negative salient terms among those who had never previously been asked to participate in research, compared to those who had previously participated. These findings are consistent with evidence suggesting that, despite decades of NIH policy efforts to improve representativeness in research participation, contemporary perceptions among underrepresented groups remain largely unchanged – underscoring the continued need for targeted, evidence-based recruitment strategies [7,27–34].

It is notable that several strongly negative salient terms – including ‘scary,’ ‘effort,’ and ‘lab rats’ – were salient across populations and questions. The prominence of ‘scary’ in particular – a salient term across multiple prompts and all demographic groups – suggests the possibility of fear and apprehension as a dimension of research perceptions among underrepresented populations, regardless of prior experience. Similarly, ‘effort’ and ‘lab rats’ may reflect perceived burdens and concerns about exploitation across demographic groups, and may represent important targets for culturally-responsive messaging and participation education. Taken together with the finding that only 101 individuals completed the survey across five months despite a relatively low-burden survey methodology with a $25 incentive, these persistent negative perceptions may themselves help explain the continued underenrollment of these populations in clinical research – consistent with prior work demonstrating the large-scale ineffectiveness of national diversity initiatives [5,6].

Additionally, our findings demonstrate a substantial overlap in sentiments across groups, suggesting that barriers and facilitators to research participation may not be specific to individual populations. This convergence of experiences across diverse underrepresented populations is consistent with prior systematic reviews, demonstrating that structural and systemic barriers to research participation (e.g., logistical barriers, access, unawareness) may affect multiple underrepresented groups similarly [35,36]. Community engagement has been demonstrated to improve recruitment and retention across underrepresented groups [37–40], and may help overcome the trepidation we found among participants considering research participation, particularly among those who were never previously approached to participate. Additionally, addressing structural barriers that affect all underrepresented groups (e.g., minimizing exclusion criteria, reducing transportation barriers and time commitment burdens, providing financial incentives, and allowing study procedure flexibility) may enhance participation overall [41,42].

Although shared experiences predominated, our findings also demonstrated unique concerns among each group. Most notably, ‘research misconduct’ emerged as uniquely salient among Black participants when considering being a research participant. While we do not have contextual information to understand what participants were thinking about when responding to our prompt, this may reflect both historic and contemporary mistrust of the biomedical establishment [43,44]. In addition to providing opportunities to participate in research, systemic, structural, and cultural changes are imperative to acknowledge past wrongs and their ongoing impact; build long-term, authentic partnerships with Black communities; and demonstrate that research benefits the communities of participants [7,45].

Another interesting finding was the difference in sentiments between those who had previously participated in research (less than one-third of participants), compared to those who had never previously been asked to participate (more than half of participants). Prior research experience resulted in more positive sentiments, while those who had never previously been asked to participate in research expressed more negative sentiments. Those without prior experience may have greater uncertainty and anxiety about what research participation entails. This finding is consistent with literature demonstrating that prior research participation is strongly associated with increased willingness to participate in future studies, and positive attitudes towards research [46,47]. Importantly, our findings suggest that increasing initial participation opportunities, i.e., simply asking more individuals from underrepresented groups to participate, may be one of the most effective long-term strategies for building research engagement, in part, by increasing familiarity with research in general, and specific research workflow, processes, and study teams. This finding additionally significantly strengthens the evidence base for community-engaged recruitment approaches – if a single positive research encounter can shift perceptions, then proactive community outreach, peer navigation, and embedded research partnerships represent high-yield investments in long-term participation representativeness.

Several limitations should be considered when interpreting our findings. First, freelisting has inherent limitations. It may capture what comes immediately to mind, but it may not reflect deeper perspectives that might emerge through qualitative interviews. Additionally, while validated for web-based administration [24], surveys may not fully capture the same contextual information that interviews with probing may. Second, our study was conducted in specific geographic regions, which may not represent the experiences of underrepresented populations across the United States. Additionally, we recruited participants primarily through healthcare settings. Individuals who regularly access healthcare and are able to navigate an online survey platform may have different perspectives on research participation than those with limited access to healthcare and online engagement. In addition, although our study examined several important underrepresented populations, we did not have ample sample size to evaluate intersectional characteristics, and did not collect socioeconomic status, educational attainment, or other pertinent demographic characteristics. These factors are recognized barriers to research participation that may interact with the demographic characteristics we examined [35,36]; future work among larger, more heterogeneous cohorts is therefore needed to contextualize our findings within the broader landscape of structural barriers to participation. Third, the example freelisting question that we provided in the survey instructions was intentionally unrelated to research, but involved a ‘scary movie’ topic, including example response terms such as ‘scary,’ afraid,’ and ‘having nightmares;’ this may have primed participants to generate fear-related terms, potentially contributing to the salience of ‘scary’ across populations and prompts. Fourth, our study design was cross-sectional, but attitudes towards research participation may change over time. Finally, it is worth noting that our data were collected between May and September 2023, a period preceding the 2025 presidential inauguration and the subsequent policy changes affecting federal support for diversity, equity, and inclusion initiatives in research. These policy shifts and subsequent public discourse may have meaningfully altered attitudes towards research participation among underrepresented populations.

## Conclusions

Leveraging freelisting, we demonstrated that historically underrepresented populations have positive and negative feelings about participating in research, with more negative salient terms among those who had never previously been asked to participate. These findings suggest that proactive outreach to individuals who have never previously been approached, combined with efforts to address persistent negative perceptions such as fear and perceived burden, may be among the most impactful strategies for improving research representativeness. Future work is needed, such as in-depth interviews or concept mapping, to understand the contextual information surrounding the sentiments we found, and to elucidate the mechanisms underlying these sentiments, ultimately enabling the development of more effective, culturally-responsive recruitment and retention strategies across diverse groups.

## Supporting information

S1 Appendix Survey. (PDF)

S2 Appendix Data dictionary. (PDF)

S3 Appendix Think about the term ‘research.’ What are all the words and phrases that you think of? (XLSX)

S4 Appendix Think about times you were asked to participate in research. Type all the words and phrases to describe how it made you feel. (XLSX)

S5 Appendix Think about being a participant in a research study. What are all the words or phrases that come to mind? (XLSX)
