# Supplementary material for: Perceptions of research participation among underrepresented groups: Insights using freelisting methodology
Source: PLoS One. 2026 Jul 1;21(7):e0351215. doi: 10.1371/journal.pone.0351215 (PMC13322552; doi:10.1371/journal.pone.0351215)
Supplement: S1 Appendix — (PDF) [file pone.0351215.s001.pdf]

English

Consent

Thank you for your interest in sharing your perspectives about research!

We are conducting a research project to better understand:

1. What affects people's decision to participate in research; and
2. Ways to improve how we recruit people who are currently not well-represented in research.

If you self-identify with one or more of these groups, you're a good fit for this study:

- Black;
- Hispanic or Latino/a/x
- Women (cisgender and transgender); and/or
- Living in a rural area.

Below you can read the consent form for this study. At the bottom of this page, you will indicate whether you want to participate. You can also download a copy of this consent form. To do so, click here: Informed consent form

UNIVERSITY OF PENNSYLVANIA
RESEARCH PARTICIPANT
INFORMED CONSENT FORM

Protocol Title:

Identifying facilitators and barriers of diversity and representativeness in randomized clinical trials

(BETTER Project 1)

Principal Investigators:
Meghan B. Lane-Fall, MD, MSHP, FCCM
meghan.lanefall@pennmedicine.upenn.edu
Rachel Kohn, MD, MSCE
rachel.kohn2@pennmedicine.upenn.edu

Additional Contact:
Adina Lieberman, MPH
adina.lieberman@pennmedicine.upenn.edu

I agree to take part in this study.

- ○ Yes, I agree to participate.
- ○ No, I do not agree to participate.

Because you selected no, you will not be enrolled in the study.

Demographic Screener

How old are you?

____

Which best describes your gender identity?

NOTE: "Trans" a.k.a. "Transgender" is an umbrella term that refers to people whose gender identity, expression, or behavior is different from those typically associated with their assigned sex at birth.

- ○ Male
- ○ Female
- ○ Non-binary/third gender
- ○ Trans male/trans man

- ○ Trans female/trans woman
- ○ [ ] Prefer to self-describe
- ○ Prefer not to say

Do you identify as Hispanic or Latino/a/x?

- ○ Yes
- ○ No
- ○ Prefer not to say

What race(s) do you identify as? (Check all that apply.)

- ☐ American Indian or Alaska Native
- ☐ Asian
- ☐ Black or African American
- ☐ Native Hawaiian or Other Pacific Islander
- ☐ White
- ☐ [ ] Other
- ☐ Prefer not to say

What 5 digit zip code do you live in?

[ ]

Do you consider where you live a rural area?

- ○ Yes
- ○ No

Have you ever been asked to participate in a research study?

- ○ Yes
- ○ No
- ○ Prefer not to say

Have you ever participated in a research study before?

- Yes
- No
- Prefer not to say

Contact Info for ineligible

Thank you for your interest in our study! Based on your responses, you are not eligible to complete our survey at this time.

Would you be interested in being contacted about future research for this study?

- Yes
- No

First Name

Last Name

Email Address

Phone Number (Please use the format: XXX-XXX-XXXX)

Survey Questions - Only for the Eligibles

First, we want to ask you questions using a tool called freelisting. **Freelisting helps us understand how different groups of people think about a topic**. **In this survey we will ask you to list words for different topics about research.**

Here is how freelisting works: We will ask you a question about a research topic.

- For each topic, type words and phrases as soon as you think of them.
- Type ALL the words and phrases you can think of.
- Type all the words or phrases in the order that you think of them.
- Do not edit the order.
- Type as many words and phrases as you want.
- There are no right or wrong answers.

Here is an example. What words and phrases do you think of when you hear “scary movie”?
You might type:

- Scared
- Afraid
- Scream
- Movie theater
- Celebrities
- Surprised
- A shocking twist
- Night
- Having nightmares
- Unable to sleep

The questions in this survey are going to ask you about participating in research studies. **Remember to type ALL the words and phrases that you think of as soon as you think of them. Please list them in the order that you think of them.**

Think about the term "research". What are all the words and phrases that you think of?

Is there anything else that comes to mind for the term “research”?

Think about times you were asked to participate* in research. Type all the words and phrases to describe how it made you feel.

*By participate, we mean sign up to be a part of a study that explores or tests new things.

Do you have any other feelings about participating in research?

Think about being a participant in a research study. What are all the words or phrases that come to mind?

Is there anything else that comes to mind when thinking about being a participant in research?

Now we want to ask you some questions about your preferences related to participating in research. You do not have to freelist your responses like in the first part of this survey.

One thing that our team is worried about is that many people do not want to participate in a research study. By participate, we mean sign up to be a part of a study that explores or tests new things. We want to hear from lots of different types of people about why they would or wouldn't want to participate in research.

Think about advice you would give us to help people agree to participate in a research study.

What are things you think we **should** do?

What are things you think we **should not** do?

Imagine someone asked you to participate in a research study.

What are the things that would make you **agree** to participate?

What are things that would make you **not agree** to participate?

How do you prefer being contacted to participate in a research study?

Here are some ways we contact people to participate in a research study.

Would you be okay with someone contacting you to participate in a research study these ways? Please check all that apply:

**NOTE: An online patient portal is a website that is available through your health system. On the online portal, you can view things like upcoming appointments, lab results, summaries from your appointments, and medication lists.*

- ☐ Phone call
- ☐ Text message
- ☐ Email

- ☐ Mailed letter
- ☐ In-person (ex. at your medical appointment)
- ☐ Message in your online patient portal (ex. MyChart)*
- ☐ [ ] Other (fill in text box)

You checked “in-person” above. That means you would be okay if someone approached you in-person about participating in a research study. Where would you feel most comfortable being approached in-person about participating in a research study? Please check all that apply:

- ☐ In the waiting room of your provider’s office
- ☐ During your appointments in the room with the provider (ex. your doctor, your nurse, your nurse practitioner, your physician assistant)
- ☐ In a community space (ex. church, community center, store)
- ☐ [ ] Other (fill in text box)

I would like the person who asks me to participate in research be similar to me in the following ways:

- ☐ Race
- ☐ Ethnicity
- ☐ Gender
- ☐ Locality (ex. living close to where you live)
- ☐ Language
- ☐ [ ] Other (fill in text box)
- ☐ The person does not need to be similar to me

Who do you prefer to ask you to participate in a research study? Ignore how similar or different they are (or seem to be) from you.

- ☐ Research staff
- ☐ Healthcare staff (ex. people who work at the front desk, medical assistants)
- ☐ Your regular healthcare provider (ex. your doctor, your nurse, your nurse practitioner, your physician assistant)
- ☐ A healthcare provider that is NOT your healthcare provider

- ☐ Community member
- ☐ [ ] Other (fill in text box)
- ☐ I do not have a preference.

Participants are often paid for their time when participating in research. How would being paid for your time impact your decision to participate or not?

[ ]

How should researchers decide who to pay for participating in research and how much to pay them?

[ ]

Sometimes, researchers will offer support to make it easier to participate in a research study. This may be instead of or in addition to paying you. For example, researchers may:

- Provide transportation, childcare, meals, and/or material prizes (ex. iPads);
- Let you choose times that are most convenient for your schedule; and/or
- Offer you the option to participate virtually/online.

What type(s) of support would make it more likely that you participate in a research study? (Do not include money.)

- ☐ Bus pass, cab voucher, or gas voucher
- ☐ Early morning/evening/night/weekend study visits to accommodate your schedule
- ☐ Meals
- ☐ Childcare
- ☐ Social recognition (ex. certificate of completion, thank you card)

- ☐ Receiving study results
- ☐ Material prizes (ex. iPads)
- ☐ [ ] Other (fill in text box)

Is there anything else about research or being asked to participate in a research study that you want us to know?

[ ]

Incentive - only for the Eligibles

As a thank you for your time and invaluable feedback, we would like to send you $25.

Would you like to receive $25?

- ○ No
- ○ Yes

To receive your $25, complete the information below:

How would you like to receive this payment?

- ○ Venmo
- ○ PayPal
- ○ Zelle
- ○ CVS gift card
- ○ Wawa gift card

Enter the email address associated with the payment method you selected above. Double check this! This is where we send your money.

Enter the phone number associated with the payment method you selected above. Double check this! This is where we send your money.

Powered by Qualtrics
