# Supplementary material for: Perceptions of research participation among underrepresented groups: Insights using freelisting methodology
Source: PLoS One. 2026 Jul 1;21(7):e0351215. doi: 10.1371/journal.pone.0351215 (PMC13322552; doi:10.1371/journal.pone.0351215)
Supplement: S2 Appendix — (PDF) [file pone.0351215.s002.pdf]

**STUDY**	**TESTS**	**ANALYZE**
study	test	analyze
studying	testing	analytics
case study	tests	analysis
a study	lab tests	
studying with lots of questions	uring testing	
some sort of study	labs	
the systematic investigation into and study of materials and sources in order to establish facts and reach new conclusions.	blood draws	
study of something specific	checking blood and cells	
to study	testing out	
studies	test products	
Clinical Trials	scan	
investigation	screenings	
investigate		
trials		
Pharmacitical testing		
research		

projects

project
a study
new study

EXPERIMENT	SEARCH	QUESTION (asking)
experiment	search	questions
experiementation	searching	question
experiments	search for cure	questioning
control group	looking	
placebo	looking for information	
cause and effect	look up	
	looking or checking for something	
	looking for different things	
	Google	
	finding info	
	reading	
	read about it	
	fact finding	
	explore	
	exploration	
	find	

finding more information

exploring

SCIENCE	RESEARCHER	DOCTOR
science	researcher	doctor
sciences	scientist	doctors
science project	researchers	practicioners
the scientific methods	investigators	white coat
hypothesis	scientists	
Method	professor	
	team	

**TREATMENT**	**SCARY**	**RESULTS**
improved treatment	scary	finding the answers to questions
new drugs	scared	answers
therapy	afraid	answer
investigational drugs	danger	result
treatment	scream	results
advance treatments	having nightmares	results or conclusions
medication		findings
medications	risks %	reaching a conclusion
new medication	vulnerable %	outcome
side effects	negativity %	the outcome
testing different medications	pain	
drugs	can be unsettling	
new medicine	risk	
	scared to work with other classmates	
	to be honest I was kind of scared	
	intimidated	

paranoid

sometimes fear
fear at times
afraid (if medical)

and pain

triggering

TEST TUBE	ACADEMIC	OPINIONS
test tube	school	opinion
glass vile	academic	opinions
	academic activites	
	university	
	student	
	educational	
	educate	
	teaching	
	college	
	classroom	
	training	
	teach	
	Sociolgy	
	academic	

**KNOWLEDGE**	**ADVANCEMENTS**	**THINKING**
knowledge	adancement	thinking
learning	advances	thoughts
understanding	future advances	thoughtful
information	innovation	brainstorming
gather information	progress	deep thinking
finding information	improving	
new information	improving healthcare	
facts	future outcomes	
fact	discovery	
learn more	discoveries	
get to know	discover	
what something is	advancing science	
inform	I think its a way to solce medical issues and viruses	
learn	I think it’s the best to advance in science and medicine	
knowing the truth	I feel it is a big part of the science world because without research we wouldn't be able to figure the things we know about life.	
knowing	breakthrough	

informative

to learn about the research topic,

RESEARCH ANIMAL	RESEARCH MISCONDUCT	RESEARCH BENEFITS
animal	misconduct	cure
mice	people hurt by pharamecutial trials	helping others
rats	abuse of minorities	helping people
rabbit	Tuskeegee	helpful
monkeys	crime	answering questions to help sciencists
hamster	death	helpful to patients
	unethical research	life saving
	dying	rewarding
	Dead	can be reassuring
		beneficial
		healing
		healing disease
		life saving
		better life
		better quality of life
		new life perspective

part of helping

help others
cures
benefit

contribiting to improve the future of the human race
like I'm contributing and making the world better

I feel very helpful for helping people find out what it is that will help them educate themselves
To help out a good cause

beneficial for others and companies
happy to help
caring
fight for others
contriute to a better cause
helping advance science or medicine

assisting medical discoveries
like to be a part of a research if it can help others
helping,
finding a cure

**TECHNOLOGY**	**LAB RATS**	**DATA**
technology	lab rat	interesting statistics about niche or general subjects
internet	being used in expriement	stats
social media	guinea pig	statistics
online	ginny pig	data
computer	guinie pig	number
	feeling like an animal/lab rat	numbers
	Test Dummy	graphs
		charts
		tables
		evidence
		measurments
		trends

DATA COLLECTION	EFFORT	PARTICIPANTS
surveys	effort	participants
polls	long term	patient
data collection	tedious	patients
gathering evidence	long	pool of people
observe	time consuming	people
observation	a lot of work	participate
interviews	work	group
questions about my life	hard work	groups of people
questions about myself	late nights	putting people in a certain category
watching	lengthy endeavor	participating
poll	dedication	participation
questions about opinions	commitment	
answering based on experience	time	
interview	meticulous	
questionnaire	detailed	
tracking my habbits	demanding on my time	

sharing feelings and thoughts	hard
giving feedback	lengthy task<br>challenging<br>time taken out of my schedule

**LABORTORY**	**INCENTIVES**	**FUNDING**
labortory	payments	funding
lab	incentives	budget
	money	expensive
	cash	
	I need cash	
	will you get paid	
	compensation	

**OBJECTIVES**	**COMPLEX**	**SURPRISED**
objective	complex	surprise
objectives	complicated	surprised

**READING MATERIALS**	**RESEARCHER RESPONSIBILITY**	**POSITIVE FEELINGS**
paper	ethical research	happy
papers	consent	trust
book	responsibility	exciting
books	verified	interesting
textbooks	proofing	awesome
newspaper	due diligence	hope
encyclopedia	IRB	excited
library	credibility	intriguing
	sign waiver	good people
	legal	trusted
	informed consent	trustworthy
		hopeful
		good
		fun
		generally positive

thankful

impressed
great
very interesting

passion

motivated

determind

I am excited to be part of this research process,

MEDICAL	CURIOSITY	TREPIDATION
Medical	curiosity	wary
Hospital	curious	cautious
Diagnosis	with curiosity	uneasy
clinical	intriguted	skeptical
sterile	inquisitive	unsure
cancer	interest	hesitant
sick	curious!	uncertaity
Pharmacy	a lot of curiosity	insecure about what is going to happen
anatomy		Generally interested even if I am apprehensive
		worried
		nervous
		what if something goes wrong
		concerns
		concern
		nervousness
		anxiety

anxious

concerned
nerves
worry

uncertain

caution

**NEVER PARTICPATED**	**WILLING TO PARTICIAPTE**	**BEING INCLUDED**
I have never particiated	interested	A part of something
I have never particiated in any research	very interested	part of
I have never particpated before	willing to help	teamwork
I've never been asked	willing	involved
never participated	open	included
I have never		include
I have not particpated		together
I've not been part of a study		being a part of
I have never been part of a study so I didn’t know what to expect		join in
		It made me feel as if I was being part of a study
		cooperation
		"part of"
		be involved
		apart of something

FEELING VALUED	SHARING RESULTS	QUESTIONS ABOUT THE PROJECT
proud	will I see the outcome	why
worthy	to be able to know the results in the future	how
honored	what is the outcome	
respected	outcomes not being shared	not knowing
wanted	I'll be anxious to see the numbers	what I felt when I saw the brochure was mostly to understand and comprehend the reason for the research
needed		What are too trying to day?
it made me glad that they asked me to participate even if I didn’t want to		What the hell?
considered		What can I learn from this?
appreciated		Can I die from this?
The phrases that i used that made me feel involved in the research survey would be I feel very honored and smart to have been chosen to be apart of the research		what are we doing
and that my opinion or voice was being heard		how long
valued		how often
To participate in something it made me feel great to learn new things.		what are you looking for
useful		are there any side affect
purposeful		how many people
pride		not knowing what to expect

considering my opinion and choices

valued

know knowing what's bing investigated

not knowing the criteria for inclusion/exclusion

why?

what will be the results?

will I have the requirements for the reasearch?

who will know

what is it about

will I get hurt

what do I have to do

is it easy

what type of research?

what are the side effects

what is the intent of the study

why am I being ask

unsure what will happen to my personal data

unsure of outsome/what it wil be used for
