# Supplementary material for: Perceptions of research participation among underrepresented groups: Insights using freelisting methodology
Source: PLoS One. 2026 Jul 1;21(7):e0351215. doi: 10.1371/journal.pone.0351215 (PMC13322552; doi:10.1371/journal.pone.0351215)
Supplement: Peer review history

# Peer review: Decision Letter 0

**DOI:** 10.1371/journal.pone.0351215.r001

**Authors:** Vijayalakshmi Kakulapati

22 Apr 2026

PONE-D-26-11831

Perceptions of research participation among underrepresented groups: Insights using freelisting methodology

PLOS One

Dear Dr. Kohn,

Thank you for submitting your manuscript to PLOS ONE. After careful consideration, we feel that it has merit but does not fully meet PLOS ONE’s publication criteria as it currently stands. Therefore, we invite you to submit a revised version of the manuscript that addresses the points raised during the review process.

Please submit your revised manuscript by Jun 05 2026 11:59PM. If you will need more time than this to complete your revisions, please reply to this message or contact the journal office at plosone@plos.org. When you’re ready to submit your revision, log on to https://www.editorialmanager.com/pone/ and select the ’Submissions Needing Revision’ folder to locate your manuscript file.

Please include the following items when submitting your revised manuscript:

- A letter that responds to each point raised by the academic editor and reviewer(s). You should upload this letter as a separate file labeled ’Response to Reviewers’.
- A marked-up copy of your manuscript that highlights changes made to the original version. You should upload this as a separate file labeled ’Revised Manuscript with Track Changes’.
- An unmarked version of your revised paper without tracked changes. You should upload this as a separate file labeled ’Manuscript’.

If you would like to make changes to your financial disclosure, please include your updated statement in your cover letter. Guidelines for resubmitting your figure files are available below the reviewer comments at the end of this letter.

If applicable, we recommend that you deposit your laboratory protocols in protocols.io to enhance the reproducibility of your results. Protocols.io assigns your protocol its own identifier (DOI) so that it can be cited independently in the future. For instructions see: https://journals.plos.org/plosone/s/submission-guidelines#loc-laboratory-protocols. Additionally, PLOS ONE offers an option for publishing peer-reviewed Lab Protocol articles, which describe protocols hosted on protocols.io. Read more information on sharing protocols at https://plos.org/protocols?utm_medium=editorial-email&utm_source=authorletters&utm_campaign=protocols.

As the corresponding author, your ORCID iD is verified in the submission system and will appear in the published article. PLOS supports the use of ORCID, and we encourage all coauthors to register for an ORCID iD and use it as well. Please encourage your coauthors to verify their ORCID iD within the submission system before final acceptance, as unverified ORCID iDs will not appear in the published article. *Only*  the individual author can complete the verification step; PLOS staff *cannot*  verify ORCID iDs on behalf of authors.

We look forward to receiving your revised manuscript.

Kind regards,

Vijayalakshmi Kakulapati, Ph.D

Academic Editor

PLOS One

Journal Requirements:

When submitting your revision, we need you to address these additional requirements.

1. Please ensure that your manuscript meets PLOS ONE’s style requirements, including those for file naming. The PLOS ONE style templates can be found at

https://journals.plos.org/plosone/s/file?id=wjVg/PLOSOne_formatting_sample_main_body.pdf and https://journals.plos.org/plosone/s/file?id=ba62/PLOSOne_formatting_sample_title_authors_affiliations.pdf

2. In the online submission form, you indicated that the datasets used and/or analyzed during the current study are available from the corresponding author on reasonable request

All PLOS journals now require all data underlying the findings described in their manuscript to be freely available to other researchers, either 1. In a public repository, 2. Within the manuscript itself, or 3. Uploaded as supplementary information.

This policy applies to all data except where public deposition would breach compliance with the protocol approved by your research ethics board. If your data cannot be made publicly available for ethical or legal reasons (e.g., public availability would compromise patient privacy), please explain your reasons on resubmission and your exemption request will be escalated for approval.

3. When completing the data availability statement of the submission form, you indicated that you will make your data available on acceptance. We strongly recommend all authors decide on a data sharing plan before acceptance, as the process can be lengthy and hold up publication timelines. Please note that, though access restrictions are acceptable now, your entire data will need to be made freely accessible if your manuscript is accepted for publication. This policy applies to all data except where public deposition would breach compliance with the protocol approved by your research ethics board. If you are unable to adhere to our open data policy, please kindly revise your statement to explain your reasoning and we will seek the editor’s input on an exemption. Please be assured that, once you have provided your new statement, the assessment of your exemption will not hold up the peer review process.

4. Thank you for stating the following financial disclosure:

This project was funded by the American Heart Association Strategically Focused Research Network on the Science of Diversity in Clinical Trials (946224)

Please state what role the funders took in the study. If the funders had no role, please state: "The funders had no role in study design, data collection and analysis, decision to publish, or preparation of the manuscript."

If this statement is not correct you must amend it as needed.

Please include this amended Role of Funder statement in your cover letter; we will change the online submission form on your behalf.

5. Please include a separate caption for each figure in your manuscript.

6. Please include captions for your Supporting Information files at the end of your manuscript, and update any in-text citations to match accordingly. Please see our Supporting Information guidelines for more information: http://journals.plos.org/plosone/s/supporting-information.

[Note: HTML markup is below. Please do not edit.]

Reviewers’ comments:

Reviewer’s Responses to Questions

**Comments to the Author**

1. Is the manuscript technically sound, and do the data support the conclusions?

The manuscript must describe a technically sound piece of scientific research with data that supports the conclusions. Experiments must have been conducted rigorously, with appropriate controls, replication, and sample sizes. The conclusions must be drawn appropriately based on the data presented.

Reviewer #1: Yes

Reviewer #2: Partly

**********

2. Has the statistical analysis been performed appropriately and rigorously?

Reviewer #1: I Don’t Know

Reviewer #2: I Don’t Know

**********

3. Have the authors made all data underlying the findings in their manuscript fully available?

The PLOS Data policy requires authors to make all data underlying the findings described in their manuscript fully available without restriction, with rare exception (please refer to the Data Availability Statement in the manuscript PDF file). The data should be provided as part of the manuscript or its supporting information, or deposited to a public repository. For example, in addition to summary statistics, the data points behind means, medians and variance measures should be available. If there are restrictions on publicly sharing data—e.g. participant privacy or use of data from a third party—those must be specified.

Reviewer #1: Yes

Reviewer #2: Yes

**********

4. Is the manuscript presented in an intelligible fashion and written in standard English?

PLOS ONE does not copyedit accepted manuscripts, so the language in submitted articles must be clear, correct, and unambiguous. Any typographical or grammatical errors should be corrected at revision, so please note any specific errors here.

Reviewer #1: Yes

Reviewer #2: Yes

**********

5. Review Comments to the Author

Please use the space provided to explain your answers to the questions above. You may also include additional comments for the author, including concerns about dual publication, research ethics, or publication ethics. (Please upload your review as an attachment if it exceeds 20,000 characters)

Reviewer #1: Thank you for the opportunity to review this manuscript. This manuscript presents the results of a cross-sectional web-based free-listing survey among adults commonly underrepresented in clinical research to elicit perceptions and emotions regarding participation in research. Understanding research participation, especially among underrepresented populations is an important issue. This study is novel as it brings attention to a qualitative methodology “freelisting” not commonly used to study research participation. Overall, the manuscript is concise and very well-written. Below, I am listing a few comments to further strengthen the accuracy of the paper.

1. In the Discussion section, the authors state “These findings suggest that we, as investigators and research teams, need to enhance our efforts to recruit representative populations into our studies, thereby ensuring that all patient groups have equal opportunities to participate.7,27-33” While the very broad statement in itself is not incorrect, I think this is not something that the results suggests. This should either be removed or reworded.

2. I also think it is interesting that the authors don’t discuss some of the major negative salient terms which stood out for almost all groups and questions, including scary, effort, lab rats.

3. One of the salient terms that stood out to me was “scary”, which is a strong negative emotion, which I think is a bit surprising. Interestingly, when I looked at the example free listing question, the topic was “scary movie” and some of the example words listed were “scary”, “having nightmares”, “afraid”, “scared”. All of these exact example words could then also be found listed under the parent terms in Appendix 2. I understand that the researchers chose a topic unrelated to research, but I think it might have affected the results, and this should be acknowledged.

4. In terms of limitations, I think it would be good to highlight that the study looked at several important underrepresented populations, but the analysis did not include lower socioeconomic status. In fact, the survey did not collect information on, for example, education. I think this should be acknowledged and discussed.

5. Have the authors considered intersectionality of the included underrepresented populations? I am not sure if the n’s would be large enough but could be discussed as future direction.

Reviewer #2: This manuscript describes the use of freelisting to investigate contemporary barriers to research participation by underrepresented groups. While the use of this methodology is new to this type of investigation, the results obtained reproduce the results of past studies using other methods (many are appropriately cited). Thus, new understandings of the “mechanisms of lack of representation (awkward wording)” are not revealed. Instead, the data support the conclusion that contemporary perceptions of research participation by a small, but appropriately representative, number of individuals who are underrepresented in clinical studies remain the same decades after the pioneering studies that elucidated them.

The finding that perceptions remain the same for those with no first-hand experience with clinical research or recruitment efforts is worthy of publication. It demonstrates that despite changes in NIH policy and national efforts to improve diversity in research participation, they continue to be largely ineffective as published in PLoS medicine over a decade ago (Oh et al., 2015). This ineffectiveness limits the rigor and impact of clinical and biomedical research, and this scientific fact must be reiterated in the scientific literature to reach multiple audiences. Moreover, the somewhat novel finding in this manuscript that perceptions of research improved for underrepresented individuals with first-hand experience in a clinical study and/or recruitment encounter is significant. This result increases the evidence-base for increased recruitment efforts using community-engaged tools as discussed by the authors. Taken together, the manuscript should be revised to better highlight these conclusions from the data presented.

Other results that warrant publication are the timeliness of the work, the use of a new methodology for gaining community insight about research participation, and the comparison across populations groups. Participant responses were collected from May-September 2023, a few years after the murder of George Floyd that prompted greater attention to ‘race’ and health, and before inauguration of the current president. Thus, this cross-sectional study captures a unique period in U.S. history. It also captures perspectives and sentiments across populations who are underrepresented in research, which is somewhat novel given that most studies on this topic study one population or another.

The fact that only 101 responses were collected in 5 months-time through implementation of freelisting (a low burden activity with a $25 incentive) is a significant result because it exemplifies the outcome of continued negative perceptions towards research in 2023 at the multiple sites for the study. At these sites, the patient/client demographics should be available and the authors could estimate how many eligible individuals were reached in the 5 months of the study to obtain only 101 responses (i.e., quasi response rate). Given that freelisting appears to be a relativity low cost, low resource tool for collecting community insight, it will be interesting to see how these perceptions change after the inauguration of the new president in 2024. The authors may consider adding their expectations to the discussion section.

Finally, with respect to categorization of terms and phrases as positive, neutral, or negative it is necessary to note the following. First, it appears to be a strength of freelisting that the terms and phrases that are collected can be easily sorted. However, a framework for the sorting of terms and phrases collected in this study as positive, neutral, or negative is not provided. The authors should consider adapting the Positive and Negative Affect Schedule (PANAS) as a theoretical framework for their categorization.

**********

6. PLOS authors have the option to publish the peer review history of their article (what does this mean?). If published, this will include your full peer review and any attached files.

If you choose “no”, your identity will remain anonymous but your review may still be made public.

**Do you want your identity to be public for this peer review?**  For information about this choice, including consent withdrawal, please see our Privacy Policy.

Reviewer #1: No

Reviewer #2: No

**********

[NOTE: If reviewer comments were submitted as an attachment file, they will be attached to this email and accessible via the submission site. Please log into your account, locate the manuscript record, and check for the action link "View Attachments". If this link does not appear, there are no attachment files.]

To ensure your figures meet our technical requirements, please review our figure guidelines: https://journals.plos.org/plosone/s/figures

You may also use PLOS’s free figure tool, NAAS, to help you prepare publication quality figures: https://journals.plos.org/plosone/s/figures#loc-tools-for-figure-preparation.

NAAS will assess whether your figures meet our technical requirements by comparing each figure against our figure specifications.

# Author comment: Author response to Decision Letter 1

**DOI:** 10.1371/journal.pone.0351215.r002

11 May 2026

May 7, 2026

Dear Editors of PLoS One:

Thank you for reviewing our manuscript titled ‘Perceptions of research participation among underrepresented groups: Insights using freelisting methodology’ (manuscript ID: PONE-D-26-11831). We hope you will agree that we have addressed all of the Reviewers’ thoughtful comments.

In the following pages we provide point-by-point responses to the Reviewers’ comments, indicating how and where we have made changes to the manuscript. Page and line numbers refer to the tracked changes version.

This manuscript has not been previously published and is not under consideration in the same or substantially similar form in any other peer-reviewed media. All authors listed have contributed sufficiently to the project to be included as authors. To the best of our knowledge, no conflict of interest, financial or other, exists.

This project was funded by the American Heart Association Strategically Focused Research Network on the Science of Diversity in Clinical Trials (#946224). The funder had no role in study design, data collection and analysis, decision to publish, or preparation of the manuscript.

Please do not hesitate to contact me if I can provide any further information.

Sincerely,

Rachel Kohn, MD, MSCE

Reviewer 1’s comments:

Thank you for the opportunity to review this manuscript. This manuscript presents the results of a cross-sectional web-based free-listing survey among adults commonly underrepresented in clinical research to elicit perceptions and emotions regarding participation in research. Understanding research participation, especially among underrepresented populations is an important issue. This study is novel as it brings attention to a qualitative methodology “freelisting” not commonly used to study research participation. Overall, the manuscript is concise and very well-written. Below, I am listing a few comments to further strengthen the accuracy of the paper.

We are very appreciative of the Reviewer’s interest and suggestions for improving the manuscript.

1. In the Discussion section, the authors state “These findings suggest that we, as investigators and research teams, need to enhance our efforts to recruit representative populations into our studies, thereby ensuring that all patient groups have equal opportunities to participate.7,27-33” While the very broad statement in itself is not incorrect, I think this is not something that the results suggests. This should either be removed or reworded.

We appreciate the Reviewer pointing this out. As such, we have revised the first paragraph of the Discussion (page 11, lines 262-277) to better align with our findings:

‘In this study, we leveraged freelisting to explore emotions and perspectives on research participation among populations underrepresented in prospective research to understand how we can develop more effective, culturally-responsive recruitment and retention strategies across groups. Overall, participants had neutral feelings about the term ‘research’ itself. However, when asked about times they had been asked to participate in research, the sentiment was overwhelmingly positive across groups. When asked about being a research study participant, salient terms spanned the spectrum of sentiments from positive to neutral to negative, similarly across groups, with more negative salient terms among those who had never previously been asked to participate in research, compared to those who had previously participated. These findings are consistent with evidence suggesting that, despite decades of NIH policy efforts to improve representativeness in research participation, contemporary perceptions among underrepresented groups remain largely unchanged – underscoring the continued need for targeted, evidence-based recruitment strategies.’

2. I also think it is interesting that the authors don’t discuss some of the major negative salient terms which stood out for almost all groups and questions, including scary, effort, lab rats.

We also appreciate this point. We have added a paragraph to the Discussion (page 12, lines 279-290) based on the Reviewer’s suggestions:

‘It is notable that several strongly negative salient terms – including ‘scary,’ ‘effort,’ and ‘lab rats’ – were salient across populations and questions. The prominence of ‘scary’ in particular – a salient term across multiple prompts and all demographic groups – suggests the possibility of fear and apprehension as a dimension of research perceptions among underrepresented populations, regardless of prior experience. Similarly, ‘effort’ and ‘lab rats’ may reflect perceived burdens and concerns about exploitation across demographic groups, and may represent important targets for culturally-responsive messaging and participation education. Taken together with the finding that only 101 individuals completed the survey across five months despite a relatively low-burden survey methodology with a $25 incentive, these persistent negative perceptions may themselves help explain the continued underenrollment of these populations in clinical research – consistent with prior work demonstrating the large-scale ineffectiveness of national diversity initiatives.’

3. One of the salient terms that stood out to me was “scary”, which is a strong negative emotion, which I think is a bit surprising. Interestingly, when I looked at the example free listing question, the topic was “scary movie” and some of the example words listed were “scary”, “having nightmares”, “afraid”, “scared”. All of these exact example words could then also be found listed under the parent terms in Appendix 2. I understand that the researchers chose a topic unrelated to research, but I think it might have affected the results, and this should be acknowledged.

We appreciate the Reviewer raising this very important limitation. We had added this as a limitation in the Discussion (page 15, lines 348-353):

‘Third, the example freelisting question that we provided in the survey instructions was intentionally unrelated to research, but involved a ‘scary movie’ topic, including example response terms such as ‘scary,’ afraid,’ and ‘having nightmares’; this may have primed participants to generate fear-related terms, potentially contributing to the salience of ‘scary’ across populations and prompts.’

4. In terms of limitations, I think it would be good to highlight that the study looked at several important underrepresented populations, but the analysis did not include lower socioeconomic status. In fact, the survey did not collect information on, for example, education. I think this should be acknowledged and discussed.

We have also added this as a limitation in the Discussion (pages 14-15, lines 342-348) as recommended by the Reviewer:

‘In addition, although our study examined several important underrepresented populations, we did not have ample sample size to evaluate intersectional characteristics, and did not collect socioeconomic status, educational attainment, or other pertinent demographic characteristics. These factors are recognized barriers to research participation that may interact with the demographic characteristics we examined; future work among larger, more heterogeneous cohorts is therefore needed to contextualize our findings within the broader landscape of structural barriers to participation.’

5. Have the authors considered intersectionality of the included underrepresented populations? I am not sure if the n’s would be large enough but could be discussed as future direction.

As mentioned by the Reviewer, we did not have ample sample size to evaluate intersectional characteristics, but have now included that as a limitation and future direction in the Discussion (pages 14-15, lines 342-348):

‘In addition, although our study examined several important underrepresented populations, we did not have ample sample size to evaluate intersectional characteristics, and did not collect socioeconomic status, educational attainment, or other pertinent demographic characteristics. These factors are recognized barriers to research participation that may interact with the demographic characteristics we examined; future work among larger, more heterogeneous cohorts is therefore needed to contextualize our findings within the broader landscape of structural barriers to participation.’

Reviewer 2’s comments:

This manuscript describes the use of freelisting to investigate contemporary barriers to research participation by underrepresented groups. While the use of this methodology is new to this type of investigation, the results obtained reproduce the results of past studies using other methods (many are appropriately cited). Thus, new understandings of the “mechanisms of lack of representation (awkward wording)” are not revealed. Instead, the data support the conclusion that contemporary perceptions of research participation by a small, but appropriately representative, number of individuals who are underrepresented in clinical studies remain the same decades after the pioneering studies that elucidated them.

We are very appreciative of the Reviewer’s comments.

We agree that the phrase ‘mechanisms of lack of representation’ was awkward, and have replaced it with ‘mechanisms driving underrepresentation’ in the Abstract and Background.

The finding that perceptions remain the same for those with no first-hand experience with clinical research or recruitment efforts is worthy of publication. It demonstrates that despite changes in NIH policy and national efforts to improve diversity in research participation, they continue to be largely ineffective as published in PLoS medicine over a decade ago (Oh et al., 2015). This ineffectiveness limits the rigor and impact of clinical and biomedical research, and this scientific fact must be reiterated in the scientific literature to reach multiple audiences. Moreover, the somewhat novel finding in this manuscript that perceptions of research improved for underrepresented individuals with first-hand experience in a clinical study and/or recruitment encounter is significant. This result increases the evidence-base for increased recruitment efforts using community-engaged tools as discussed by the authors. Taken together, the manuscript should be revised to better highlight these conclusions from the data presented.

We appreciate these important points. As such, we have revised the first paragraph of the Discussion (page 11, lines 262-277), and now include the Oh et al citation suggested by the Reviewer, as well as the fifth paragraph of the Discussion (pages 13-14, lines 316-331):

‘In this study, we leveraged freelisting to explore emotions and perspectives on research participation among populations underrepresented in prospective research to understand how we can develop more effective, culturally-responsive recruitment and retention strategies across groups. Overall, participants had neutral feelings about the term ‘research’ itself. However, when asked about times they had been asked to participate in research, the sentiment was overwhelmingly positive across groups. When asked about being a research study participant, salient terms spanned the spectrum of sentiments from positive to neutral to negative, similarly across groups, with more negative salient terms among those who had never previously been asked to participate in research, compared to those who had previously participated. These findings are consistent with evidence suggesting that, despite decades of NIH policy efforts to improve representativeness in research participation, contemporary perceptions among underrepresented groups remain largely unchanged – underscoring the continued need for targeted, evidence-based recruitment strategies.’

‘Another interesting finding was the difference in sentiments between those who had previously participated in research (less than one-third of participants), compared to those who had never previously been asked to participate (more than half of participants). Prior research experience resulted in more positive sentiments, while those who had never previously been asked to participate in research expressed more negative sentiments. Those without prior experience may have greater uncertainty and anxiety about what research participation entails. This finding is consistent with literature demonstrating that prior research participation is strongly associated with increased willingness to participate in future studies, and positive attitudes towards research. Importantly, our findings suggest that increasing initial participation opportunities, i.e., simply asking more individuals from underrepresented groups to participate, may be one of the most effective long-term strategies for building research engagement, in part, by increasing familiarity with research in general, and specific research workflow, processes, and study teams. This finding additionally significantly strengthens the evidence base for community-engaged recruitment approaches – if a single positive research encounter can shift perceptions, then proactive community outreach, peer navigation, and embedded research partnerships represent high-yield investments in long-term participation representativeness.’

We have additionally changed the Conclusions section of the Abstract (page 3, lines 101-111) and the Conclusions (pages 15-16, lines 361-373) to highlight these conclusions from the data presented:

‘Conclusions: Underrepresented populations hold positive and negative views about clinical research, with more negative perceptions among those never previously approached. These findings suggest that proactive outreach to individuals who have never previously been approached, combined with efforts to address persistent negative perceptions such as fear, may be among the most impactful strategies for improving research representativeness. Future work is needed to understand the contextual information surrounding the sentiments we found, and to elucidate the mechanisms underlying these sentiments, ultimately enabling the development of more effective, culturally-responsive recruitment and retention strategies across diverse groups.’

‘Conclusions

Leveraging freelisting, we demonstrated that historically underrepresented populations have positive and negative feelings about participating in research, with more negative salient terms among those who had never previously been asked to participate. These findings suggest that proactive outreach to individuals who have never previously been approached, combined with efforts to address persistent negative perceptions such as fear and perceived burden, may be among the most impactful strategies for improving research representativeness. Future work is needed, such as in-depth interviews or concept mapping, to understand the contextual information surrounding the sentiments we found, and to elucidate the mechanisms underlying these sentiments, ultimately enabling the development of more effective, culturally-responsive recruitment and retention strategies across diverse groups.’

Other results that warrant publication are the timeliness of the work, the use of a new methodology for gaining community insight about research participation, and the comparison across populations groups. Participant responses were collected from May-September 2023, a few years after the murder of George Floyd that prompted greater attention to ‘race’ and health, and before inauguration of the current president. Thus, this cross-sectional study captures a unique period in U.S. history. It also captures perspectives and sentiments across populations who are underrepresented in research, which is somewhat novel given that most studies on this topic study one population or another.

We appreciate this poignant reflection as well. We have incorporated this into the limitation section of the Discussion (page 15, lines 354-359):

‘Finally, it is worth noting that our data were collected between May and September 2023, a period preceding the 2025 presidential inauguration and the subsequent policy changes affecting federal support for diversity, equity, and inclusion initiatives in research. These policy shifts and subsequent publi

# Peer review: Decision Letter 1

**DOI:** 10.1371/journal.pone.0351215.r003

**Authors:** Vijayalakshmi Kakulapati

26 May 2026

Perceptions of research participation among underrepresented groups: Insights using freelisting methodology

PONE-D-26-11831R1

Dear Dr. Kohn,

We’re pleased to inform you that your manuscript has been judged scientifically suitable for publication and will be formally accepted for publication once it meets all outstanding technical requirements.

Within one week, you’ll receive an e-mail detailing the required amendments. When these have been addressed, you’ll receive a formal acceptance letter and your manuscript will be scheduled for publication.

An invoice will be generated when your article is formally accepted. Please note, if your institution has a publishing partnership with PLOS and your article meets the relevant criteria, all or part of your publication costs will be covered. Please make sure your user information is up-to-date by logging into Editorial Manager at Editorial Manager® and clicking the ‘Update My Information’ link at the top of the page. For questions related to billing, please contact billing support.

If your institution or institutions have a press office, please notify them about your upcoming paper to help maximize its impact. If they’ll be preparing press materials, please inform our press team as soon as possible -- no later than 48 hours after receiving the formal acceptance. Your manuscript will remain under strict press embargo until 2 pm Eastern Time on the date of publication. For more information, please contact onepress@plos.org.

Kind regards,

Vijayalakshmi Kakulapati, Ph.D

Academic Editor

PLOS One

**Comments to the Author**

Reviewer #1: All comments have been addressed

Reviewer #2: All comments have been addressed

**********

# Editor report: Acceptance letter

**DOI:** 10.1371/journal.pone.0351215.r004

**Authors:** Vijayalakshmi Kakulapati

PONE-D-26-11831R1

PLOS One

Dear Dr. Kohn,

I’m pleased to inform you that your manuscript has been deemed suitable for publication in PLOS One. Congratulations! Your manuscript is now being handed over to our production team.

At this stage, our production department will prepare your paper for publication. This includes ensuring the following:

* All references, tables, and figures are properly cited

* All relevant supporting information is included in the manuscript submission,

* There are no issues that prevent the paper from being properly typeset

You will receive further instructions from the production team, including instructions on how to review your proof when it is ready. Please keep in mind that we are working through a large volume of accepted articles, so please give us a few days to review your paper and let you know the next and final steps.

Lastly, if your institution or institutions have a press office, please let them know about your upcoming paper now to help maximize its impact. If they’ll be preparing press materials, please inform our press team within the next 48 hours. Your manuscript will remain under strict press embargo until 2 pm Eastern Time on the date of publication. For more information, please contact onepress@plos.org.

You will receive an invoice from PLOS for your publication fee after your manuscript has reached the completed accept phase. If you receive an email requesting payment before acceptance or for any other service, this may be a phishing scheme. Learn how to identify phishing emails and protect your accounts at https://explore.plos.org/phishing.

If we can help with anything else, please email us at customercare@plos.org.

Thank you for submitting your work to PLOS ONE and supporting open access.

Kind regards,

PLOS ONE Editorial Office Staff

on behalf of

Dr. Vijayalakshmi Kakulapati

Academic Editor

PLOS One
